# KLF2 Is Associated with ERK1/2–MAP2 Activation and Neuron-like Phenotypic Remodeling of PDGFR-β-Lineage Cells Following Ischemic Stroke

**DOI:** 10.3390/brainsci16090924

**Published:** 2026-08-30

**Authors:** Qiulu Liu, Sutong Xu, Bei Zhang, Chengyu Lv, Chenming Liu, Yali Wang, Haiyue Zhou, Yuping Luo, Siguang Li

**Affiliations:** 1 Key Laboratory of Spine and Spinal Cord Injury Repair and Regeneration of Ministry of Education, Tongji Hospital, School of Medicine, Tongji University, Shanghai 200070, China; liuqiulu@alumni.tongji.edu.cn (Q.L.); 1911450@tongji.edu.cn (S.X.); 2432340@tongji.edu.cn (B.Z.); 2532065@tongji.edu.cn (C.L.); 2211409@tongji.edu.cn (C.L.); 2332209@tongji.edu.cn (Y.W.); 2332210@tongji.edu.cn (H.Z.); luoyuping@163.com (Y.L.); 2 Stem Cell Research Center, School of Medicine, Tongji University, Shanghai 200070, China

**Keywords:** pericyte, stroke, NVU, KLF2, phenotypic remodeling, ERK1/2, MAP2

## Abstract

**Highlights:**

**What are the main findings?**

- Ischemic injury induces stage-dependent changes in PDGFR-β-lineage pericytes, including increased expression of Nestin and transient neuroglial- and neuronal-associated markers such as GFAP and DCX.
- In human brain vascular pericytes, OGD/R is associated with increased expression of DCX, MAP2, and NeuN, which are attenuated by KLF2 knockdown.

**What are the implications of the main findings?**

- These findings indicate that PDGFR-β-lineage pericytes undergo dynamic phenotypic remodeling after ischemic injury, characterized in part by transient expression of neuroglial- and neuronal-associated molecular markers.
- KLF2 is associated with ERK1/2 activation and MAP2 expression during ischemia-related pericyte phenotypic remodeling, providing a basis for further investigation of the molecular regulation of these neuron-like changes.

**Abstract:**

**Background/Objectives:** Ischemic stroke (IS) induces substantial phenotypic remodeling within the neurovascular unit, and pericytes have been implicated in the cellular responses to ischemic injury. However, the molecular characteristics and regulatory mechanisms underlying pericyte phenotypic remodeling after ischemic stroke remain incompletely understood. **Methods:** In this study, we used PDGFR-β-CreERT2; ZsGreen lineage-tracing mice and a middle cerebral artery occlusion (MCAO) model to characterize the temporal changes in PDGFR-β-lineage cells following ischemic injury. In vitro, human brain vascular pericytes (HBVPs) exposed to oxygen–glucose deprivation/reperfusion (OGD/R) and subsequently maintained in neurobasal medium to examine ischemia-related phenotypic changes. The involvement of KLF2-associated signaling was further investigated using KLF2 knockdown approaches. **Results:** We observed increased expression of Nestin in PDGFR-β-lineage cells during the early post-ischemic period, followed by the emergence of subsets co-expressing GFAP or DCX during the subacute stage. These findings indicate the acquisition of neuroglial- and neuronal-associated molecular features, rather than providing definitive evidence of lineage conversion or functional differentiation. The expression of these markers declined at later stages, whereas PDGFR-β-lineage cells were also associated with vascular remodeling during the recovery phase. In vitro, OGD/R-treated HBVPs exhibited increased expression of DCX, MAP2, and NeuN. KLF2 knockdown attenuated these molecular changes. Consistently, OGD/R was associated with increased KLF2, phosphorylated ERK1/2, and MAP2 expression, whereas KLF2 knockdown reduced ERK1/2 phosphorylation and MAP2 expression. These findings suggest that KLF2 is associated with ERK1/2 activation and MAP2 expression during ischemia-related phenotypic remodeling of PDGFR-β-lineage cells. **Conclusions:** Overall, our results identify transient neuroglial- and neuronal-associated molecular changes in pericytes after ischemic injury and provide evidence for an association between KLF2 and ERK1/2–MAP2 signaling in this process, while further studies are required to determine whether these changes represent stable lineage conversion or functional neuronal differentiation.

## 1. Introduction

Ischemic stroke (IS) is one of the leading causes of death and long-term neurological disability worldwide [1]. Its core pathological processes include interruption of cerebral blood flow, imbalance of energy metabolism, disruption of the neurovascular unit (NVU), and secondary inflammatory responses [2]. Although acute reperfusion therapy has significantly improved the prognosis of some patients, most patients still suffer from varying degrees of neurological deficits. Recent studies suggest that post-stroke tissue repair does not solely depend on neurons themselves but rather involves coordinated remodeling among multiple NVU components, including neurons, glial cells, vascular endothelial cells, and pericytes [3,4]. Pericytes are important mural cells that surround the abluminal surface of microvascular endothelial cells and play critical roles in maintaining blood–brain barrier (BBB) integrity, regulating cerebral blood flow, stabilizing capillary structure, and preserving neurovascular homeostasis [5]. In recent years, accumulating evidence has demonstrated that brain pericytes not only participate in inflammatory regulation [6], vascular repair, and fibrotic responses after injury [7], but also exhibit stem cell-associated or lineage-associated molecular features under specific injury or culture conditions [8,9,10,11,12]. However, whether these observations represent stable multilineage differentiation or instead reflect context-dependent phenotypic remodeling remains under debate. In particular, lineage-tracing studies have challenged the interpretation of endogenous pericytes as broadly multipotent stem cells in vivo [13]. Thus, characterization of pericyte responses after ischemic injury requires careful distinction between the acquisition of lineage-associated molecular markers and definitive lineage conversion.

Krüppel-like factor 2 (KLF2) is a highly conserved zinc-finger transcription factor that plays essential roles in maintaining vascular endothelial homeostasis [14], regulating inflammatory responses [15], preserving BBB integrity [16,17,18], and conferring neuroprotection following cerebral ischemia [19,20]. KLF2 has also been associated with cellular differentiation and neural-related processes in several experimental contexts [21]. However, whether KLF2 is involved in the phenotypic remodeling of pericytes following ischemic stress remains unclear. Importantly, the relationship between KLF2 expression and the acquisition of neuronal-associated molecular features in pericytes has not been established.

The extracellular signal-regulated kinase (ERK) pathway is a major intracellular signaling cascade involved in cell proliferation, differentiation, stress responses, and cell-state regulation [22,23]. ERK1/2 signaling has also been implicated in neuronal development, neuronal maturation, and post-injury neural plasticity [24,25,26]. Moreover, astrocyte activation and neuroplasticity following IS are closely linked to ERK signaling [27]. Because ERK1/2 activity can influence cell-state transitions in response to environmental stimuli, it may represent a potential signaling pathway associated with phenotypic changes in pericytes after ischemic injury. However, whether ERK1/2 signaling is associated with the acquisition of neuronal-associated molecular features in pericytes after ischemic stress remains unclear. To address this question, we used PDGFR-β-CreERT2; ZsGreen lineage-tracing mice subjected to middle cerebral artery occlusion (MCAO) to characterize the temporal changes in PDGFR-β-lineage cells following ischemic injury. We further established an oxygen–glucose deprivation/reperfusion (OGD/R) model using human brain vascular pericytes (HBVPs) to examine ischemia-related changes in neuronal-associated molecular markers and to investigate the association of KLF2 with ERK1/2 activation and MAP2 expression. Together, these approaches were used to characterize the phenotypic remodeling of PDGFR-β-lineage cells after ischemic injury and to explore potential molecular associations involving KLF2 and ERK1/2–MAP2 signaling.

## 2. Materials and Methods

### 2.1. Establishment of the MCAO Model in Pericyte Lineage-Tracing Mice and Collection of Brain Tissue Samples

PDGFR-β-CreERT2 mice (The Jackson Laboratory, Bar Harbor, ME, USA) were crossbred with ZsGreen reporter mice to generate PDGFR-β-CreERT2; ZsGreen lineage-tracing mice. These lineage-tracing mice were generated on a C57BL/6 genetic background. All experimental animals used in this study were male mice at 8 weeks of age, with body weights ranging from 20 to 25 g at the beginning of the experiments. The animals were randomly assigned to the experimental groups (MCAO groups at different time points after ischemic injury). Animals were housed under standard laboratory conditions with controlled temperature and humidity and a 12 h light/dark cycle. For lineage tracing, a 1% (*w*/*v*) tamoxifen solution was prepared in a vehicle consisting of corn oil and absolute ethanol (9:1, *v*/*v*). Corn oil and ethanol were preheated to 37 °C and 55 °C, respectively, before mixing. PDGFR-β-CreERT2; ZsGreen mice received intraperitoneal injections of tamoxifen (200 μL per mouse) once daily for five consecutive days. Following a 7-day washout period to allow Cre-mediated recombination and clearance of residual tamoxifen, mice were subjected to MCAO surgery.

Mice were anesthetized with tribromoethanol under appropriate monitoring, and transient focal cerebral ischemia was induced by MCAO. Briefly, a filament was introduced into the middle cerebral artery and maintained for 30 min before withdrawal to allow reperfusion. Postoperative supportive care included maintenance of body temperature during recovery, subcutaneous administration of sterile saline to support fluid balance, and provision of nutritional jelly to facilitate postoperative food intake. Animals were closely monitored during the postoperative period for general condition, mobility, food intake, and signs of distress. Animals that died during the surgical or immediate postoperative period or experienced technical failure during MCAO were excluded. At 1, 3, 7, 14, and 28 days after MCAO, mice were deeply anesthetized and transcardially perfused. Brains were collected, post-fixed overnight in 4% paraformaldehyde, cryoprotected sequentially in 20% and 30% (*w*/*v*) sucrose solutions, embedded in OCT compound, and stored at −80 °C. Coronal sections spanning the ischemic area (Bregma +1.10 mm to +0.98 mm) were collected using a cryostat and mounted onto glass slides for subsequent immunofluorescence staining and imaging. Each section was cut at a thickness of 10 μm, and serial sections were collected at 50 μm intervals (one section collected every five consecutive sections) for quantitative analysis.

### 2.2. LSCI and TTC Staining

To validate the success of MCAO, cerebral blood flow (CBF) was monitored using laser speckle imaging (LSCI; RFLSI ZW, RWD Life Science, China) before MCAO, during the 30 min occlusion period, and after reperfusion. CBF values were normalized to the pre-ischemic baseline and expressed as a percentage of baseline CBF. Successful MCAO was defined as a reduction in regional CBF to <20% of the pre-ischemic baseline during the 30 min occlusion period, corresponding to a reduction of >80% from baseline. Following removal of the filament and reperfusion, CBF recovered to >70% of the pre-ischemic baseline, confirming restoration of cerebral perfusion.

TTC (2, 3, 5-Triphenyltetrazolium chloride) staining was performed to confirm the presence of ischemic brain injury after MCAO. Mice underwent 30 min of middle cerebral artery occlusion followed by reperfusion, and fresh brain tissues were collected 24 h after reperfusion. Brains were sectioned into 2 mm coronal slices and incubated with 1% TTC solution at 37 °C for 20 min in the dark. White areas indicate ischemic injury regions, whereas red areas represent non-injured tissues. In the present study, TTC staining was used as a qualitative confirmation of the presence and anatomical distribution of the ischemic lesion and was not used as a quantitative endpoint for infarct area or infarct volume analysis. In these two experiments, 8-week-old male C57BL/6 mice were subjected to MCAO.

### 2.3. In Vitro Pericyte Culture and Lentiviral Infection

Human brain vascular pericytes (HBVPs; IMMOCELL Biotechnology Co., Ltd., Shenzhen, China, IM-H711) were maintained in HBVP-specific culture medium (IMMOCELL, IM-H711-1) at 37 °C in a humidified atmosphere containing 5% CO_2_. The medium was refreshed every 2 days. Upon reaching approximately 80% confluence, cells were dissociated with 0.25% trypsin and allocated to either the sh-NC group (non-targeting control shRNA. Control seq: 5′ TTCTCCGAACGTGTCACGT 3′) or the shKLF2 group (*KLF2*-targeting shRNA. Target seq: shKLF2(#138): 5′ AGTTCGCATCTGAAGGCGCAT 3′; shKLF2(#139): 5′ CGCCTTTCGGTGGCCCTGGTT 3′; shKLF2(#140): 5′ CGGCACCGACGACGACCTCAA 3′). Lentiviral vectors and packaging plasmids were obtained from Shanghai GeneChem Co., Ltd. (Shanghai, China). HBVPs were seeded into six-well plates 24 h before transduction and subsequently infected with the corresponding lentiviral constructs. The medium was replaced 12 h after infection, and cells were harvested 48 h later for downstream analyses.

### 2.4. Establishment of the OGD/R Model In Vitro

To establish the OGD/R model, HBVPs were seeded into six-well plates and cultured until approximately 70% confluence. The culture medium was then replaced with glucose-free DMEM (Gibco, Grand Island, NY, USA, 11966-025). Cells were subjected to OGD by incubation in a sealed anaerobic chamber containing an AnaeroPack oxygen-depleting system (Mitsubishi Gas Chemical, Tokyo, Japan, C-01/C-31) for 1 h. Following OGD, cells were returned to normoxic conditions and cultured in Neurobasal™-A medium (Gibco, 10888-022) supplemented with 2 mM GlutaMAX™-I (Gibco, 35050061), 2% B27 (Beyotime, Shanghai, China, C0349), and 1% penicillin–streptomycin (Thermo, Waltham, MA, USA, 10378016). Cells were maintained under these conditions for 14 days before subsequent analyses. Ctrl groups were maintained in the same Neurobasal-A/B27/GlutaMAX culture medium and for the same duration as the OGD/R groups, except that they were not subjected to OGD.

### 2.5. Immunofluorescence Staining and Imaging

Mouse brain sections were blocked with QuickBlock™ Blocking Buffer for Immunol Staining (Beyotime Biotechnology, Shanghai, China, P0260) for 1 h. Primary antibodies were diluted in Immunol Staining Primary Antibody Dilution Buffer (Beyotime Biotechnology, P0103) at the indicated ratios and incubated with the sections overnight at 4 °C. Cultured HBVPs were grown on coverslips, fixed with 4% PFA for 15 min, and permeabilized with 0.3% Triton X-100 (Solarbio, Beijing, China, T8200) for 15 min. Cells were then blocked with 3% BSA at room temperature for 1 h, followed by incubation with primary antibodies overnight at 4 °C. On the following day, both mouse brain sections and cultured cells were incubated with secondary antibodies and DAPI (BD Pharmingen, San Diego, CA, USA, 564907, 1:1000) for 1 h at room temperature. Brain sections and cells were mounted using ProLong™ Diamond (Invitrogen, Carlsbad, CA, USA, P36970) and coverslips. The following primary antibodies were used: anti-Nestin (Abcam, Cambridge, UK, ab6142, 1:200), anti-GFAP (ABclonal, Wuhan, China, A0237, 1:400), anti-Doublecortin (Abcam, ab18723, 1:500), anti-CD31 (Invitrogen, PA5-32321, 1:100), anti-PDGFR-β (Abcam, ab32570, 1:100), anti-MAP2 (Cell Signaling Technology, Danvers, MA, USA, 8707, 1:200), anti-NeuN (Abcam, ab104224, 1:500), Donkey anti-Rabbit Alexa Fluor™555 (Invitrogen, A-31572, 1:1000), and Donkey anti-Mouse Alexa Fluor™647 (Invitrogen, A-31571, 1:1000).

Immunofluorescence-stained brain sections and cell coverslips were imaged using a Leica SP8 confocal laser scanning microscope (Leica Microsystems, Wetzlar, Germany). For in vivo brain tissue analysis, images were acquired from anatomically matched regions across experimental groups using identical imaging parameters, including laser power, gain, and exposure settings. Multiple predefined fields of view were randomly selected from each section, and multiple sections were analyzed for each animal (at least three slices were taken of each animal, and three fields of view were selected for each slice during imaging). Quantitative measurements obtained from individual fields or sections were averaged within each animal before statistical analysis. Therefore, the animal, rather than an individual cell, field, or section, was considered the biological experimental unit for all in vivo analyses. For in vitro immunofluorescence analysis, multiple fields were selected from each coverslip, and multiple coverslips or independent experiments were evaluated. Measurements from individual fields were averaged within each independent biological replicate before statistical analysis. Thus, *n* represents the number of independent biological experiments, and technical measurements within each replicate were averaged prior to statistical analysis.

### 2.6. Western Blot

Collected cell samples were lysed on ice for 20 min using RIPA lysis buffer supplemented with phosphatase inhibitor (Beyotime Biotechnology, P0013B). The lysates were centrifuged at 12,000× *g* for 10 min at 4 °C, and protein concentrations were determined using the Enhanced BCA Protein Assay Kit (Beyotime Biotechnology, P0010S). Proteins were subsequently separated by 8% SDS-polyacrylamide gel electrophoresis (8% SDS-PAGE) and transferred onto membranes. After blocking with 5% non-fat milk for 1 h at room temperature, the membranes were incubated with primary antibodies overnight at 4 °C. The following day, the membranes were washed with TBST at room temperature and incubated with horseradish peroxidase (HRP)-conjugated anti-rabbit IgG or anti-mouse IgG secondary antibodies (ABclonal, 1:5000) for 1 h at room temperature. The membranes were then washed three times with TBST for 5 min each. Protein bands were visualized by immersion in ECL detection reagent (Biosharp, Hefei, China, BL520A) at room temperature and imaged using a ChemiDoc™ imaging system (Bio-Rad Laboratories, Hercules, CA, USA). The primary antibodies used were as follows: anti-KLF2 (Abcam, ab203591, 1:500), anti-Phospho-ERK1/2 (ABclonal, AP0472, 1:1000), anti-ERK1/2 (ABclonal, A10613, 1:1000), anti-MAP2 (Cell Signaling Technology, 8707, 1:1000), anti-PDGFR-β (Abcam, ab32570, 1:2000), and anti-GAPDH (Proteintech, Rosemont, IL, USA, 60004-1-Ig, 1:10,000).

### 2.7. RT-qPCR

Total RNA was extracted from cells using RNAiso Plus (Takara, Kusatsu, Shiga, Japan, 9109) according to the manufacturer’s instructions. Reverse transcription was performed using HiScript III RT SuperMix for qPCR (Vazyme, Nanjing, China, R323-01). Quantitative PCR was conducted using ChamQ Universal SYBR qPCR Master Mix (Vazyme, Q711-02) and gene-specific primers (*KLF2*-F: 5′-CCAAGAGTTCGCATCTGAAGGC-3′, *KLF2*-R: 5′-CCGTGTGCTTTCGGTAGTGGC-3′; *ACTB*-F: 5′-CACCATTGGCAATGAGCGGTTC-3′, *ACTB*-R: 5′-AGGTCTTTGCGGATGTCCACGT-3′; *GAPDH*-F: 5′- GTCTCCTCTGACTTCAACAGCG-3′, *GAPDH*-R: 5′- ACCACCCTGTTGCTGTAGCCAA-3′). *KLF2* mRNA expression was quantified using the 2^−ΔΔCt method and normalized independently to *ACTB* and *GAPDH*. Relative *KLF2* expression was calculated separately using each reference gene, and the consistency of the results was assessed between the two normalization approaches.

### 2.8. Statistical Analysis

Data are presented as individual data points with mean ± 95% confidence interval (CI), unless otherwise indicated. Measurements from multiple microscopic fields obtained from the same mouse were averaged to generate one value per animal. Thus, the individual mouse was considered the experimental unit, and multiple fields from the same animal were not treated as independent biological replicates.

Comparisons between the ipsilateral and contralateral regions obtained from the same animals were performed using two-tailed paired Student’s *t*-tests. Comparisons between two independent groups were performed using two-tailed unpaired Student’s *t*-tests. When the assumption of equal variances was not met, Welch’s correction was applied. For comparisons involving more than two groups with one independent factor, one-way ANOVA followed by the prespecified multiple-comparisons test was used. For experiments involving two independent factors, two-way ANOVA followed by Sidak’s multiple-comparisons test was used.

For proportion-based outcomes, positive and total cell counts were analyzed using binomial or beta-binomial regression, as appropriate, rather than treating percentages as unrestricted continuous variables. Beta-binomial models were used when additional dispersion beyond the binomial assumption was present. The individual mouse was treated as the experimental unit. Model-estimated proportions and between-group differences were reported with corresponding 95% CIs. For multiple pairwise comparisons in proportion-based analyses, *p* values were adjusted using the Holm method.

For parametric analyses, normality was assessed using the Shapiro–Wilk test and homogeneity of variance was evaluated using the Brown–Forsythe test. Statistical analyses and graph generation were performed using GraphPad Prism 8.0.2. Individual data points and 95% CIs are shown to provide information on variability and estimation precision. Statistical significance was defined as follows: ns, *p* ≥ 0.05; * *p* < 0.05; ** *p* < 0.01; *** *p* < 0.001 and **** *p* < 0.0001.

## 3. Results

### 3.1. Ischemic Injury Induces Temporal Changes in Nestin Expression in PDGFR-β-Lineage Cells In Vivo

**Figure 1:**
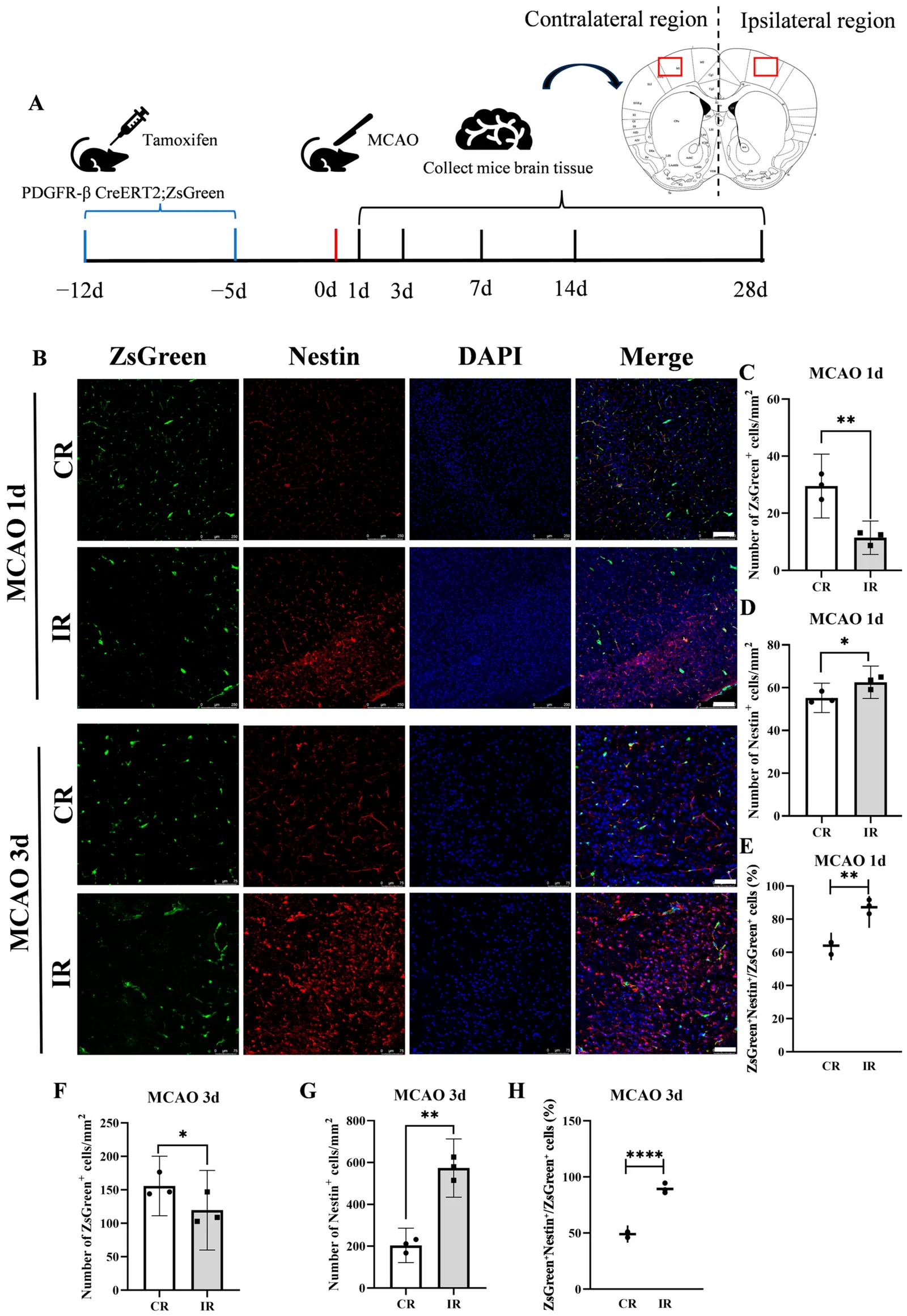
Ischemic injury induces temporal changes in Nestin expression in PDGFR-β-lineage cells in the mouse brain. (**A**) Schematic illustration of the experimental design and brain sample collection from MCAO-treated PDGFR-β-CreERT2; ZsGreen lineage-tracing mice. The red boxes indicate the regions selected for immunofluorescence imaging. Ischemic Region: ischemic injury area of cerebral cortex; Contralateral Region: contralateral cerebral cortex non-ischemic injury area. (**B**) Representative immunofluorescence images showing ZsGreen and Nestin expression in brain sections from mice at 1 and 3 days after MCAO. ZsGreen labels PDGFR-β-lineage cells. CR: Contralateral Region; IR: Ischemic Region. Color channels are indicated as follows: green, ZsGreen; red, Nestin; and blue, DAPI. Scale bars (White thick line): 75 μm. (**C**–**E**) Quantification of ZsGreen-positive cells and Nestin-positive cells in MCAO 1d mice shown in (**B**). (**C**) Quantification of number of ZsGreen^+^ cells per mm^2^; t_(2)_ = 10.84, *p* = 0.0084, mean difference = −18.08, 95% CI [−25.25, −10.90]. (**D**) Quantification of number of Nestin^+^ cells per mm^2^; t_(2)_ = 4.80, *p* = 0.0408, mean difference = 7.301, 95% CI [0.76, 13.84]. (**E**) Quantification of the percentage of ZsGreen^+^Nestin^+^ cells among ZsGreen^+^ cells. Proportion data were analyzed using binomial regression, with the individual mouse considered the experimental unit. Data were presented as model-estimated proportions with 95% confidence intervals. Estimated difference = 23.23 percentage points, 95% CI [10.51, 35.96], *p* = 0.0022. (**F**–**H**) Quantification of ZsGreen-positive cells and Nestin-positive cells in MCAO 3d mice shown in (**B**). (**F**) Quantification of number of ZsGreen^+^ cells per mm^2^; t_(2)_ = 8.49, *p* = 0.0136, mean difference = −36.27, 95% CI [−54.66, −17.89]. (**G**) Quantification of number of Nestin^+^ cells per mm^2^; t_(2)_ = 10.96, *p* = 0.0082, mean difference = 369.6, 95% CI [224.50, 514.70]. (**H**) Quantification of the percentage of ZsGreen^+^Nestin^+^ cells among ZsGreen^+^ cells. Proportion data were analyzed using binomial regression, with the individual mouse considered the experimental unit. Data were presented as model-estimated proportions with 95% confidence intervals. Estimated difference = 40.29 percentage points, 95% CI [30.78, 49.79], *p* < 0.0001. Data are presented as individual mice with mean ± 95% CI (*n* = 3 mice per time point). Multiple microscopic fields from each mouse were averaged to obtain one value per animal and were not treated as independent biological replicates. Because the IR and CR were obtained from the same animals, comparisons between the two regions (number of cells per mm^2^) were performed using a two-tailed paired Student’s *t*-test. * *p*  <  0.05, ** *p*  <  0.01, **** *p*  <  0.0001.

To characterize the temporal response of PDGFR-β-lineage cells to ischemic injury, tamoxifen-induced PDGFR-β-CreERT2; ZsGreen lineage-tracing mice were subjected to MCAO, and brain tissues were collected at the indicated time points after ischemia (Figure 1A). Quantitative analysis showed that the number of ZsGreen^+^ cells in the ischemic region (IR) was approximately half of that observed in the corresponding contralateral region (CR) at 1 day after MCAO (Figure 1B,C). In contrast, the number of Nestin^+^ cells was higher in the IR than in the CR (Figure 1B,D). Furthermore, approximately 80% of ZsGreen^+^ cells co-expressed Nestin in the IR, which was significantly higher than in the CR (Figure 1B,E). These findings demonstrate an early increase in Nestin expression among PDGFR-β-lineage cells following ischemic injury.

At 3 days after MCAO, ZsGreen^+^ cell numbers in the IR were reduced by approximately one-third compared with the CR (Figure 1B,F). The number of Nestin^+^ cells in the IR at 3 days after MCAO was approximately three times higher than that in the CR (Figure 1B,G). Compared to the IR at 1 day after MCAO, Nestin^+^ cells in the IR increased by nearly ninefold at 3 days after MCAO. Consistently, the proportion of ZsGreen^+^ cells co-expressing Nestin was significantly higher in the IR than in the CR (Figure 1B,H). Together, these results indicate that ischemic injury is associated with increased Nestin expression among PDGFR-β-lineage cells during the early post-ischemic period, with the proportion of Nestin-expressing lineage-traced cells remaining elevated at 3 days after MCAO.

### 3.2. PDGFR-β-Lineage Cells Exhibit Transient GFAP- and DCX-Associated Molecular Changes After Ischemic Injury

**Figure 2:**
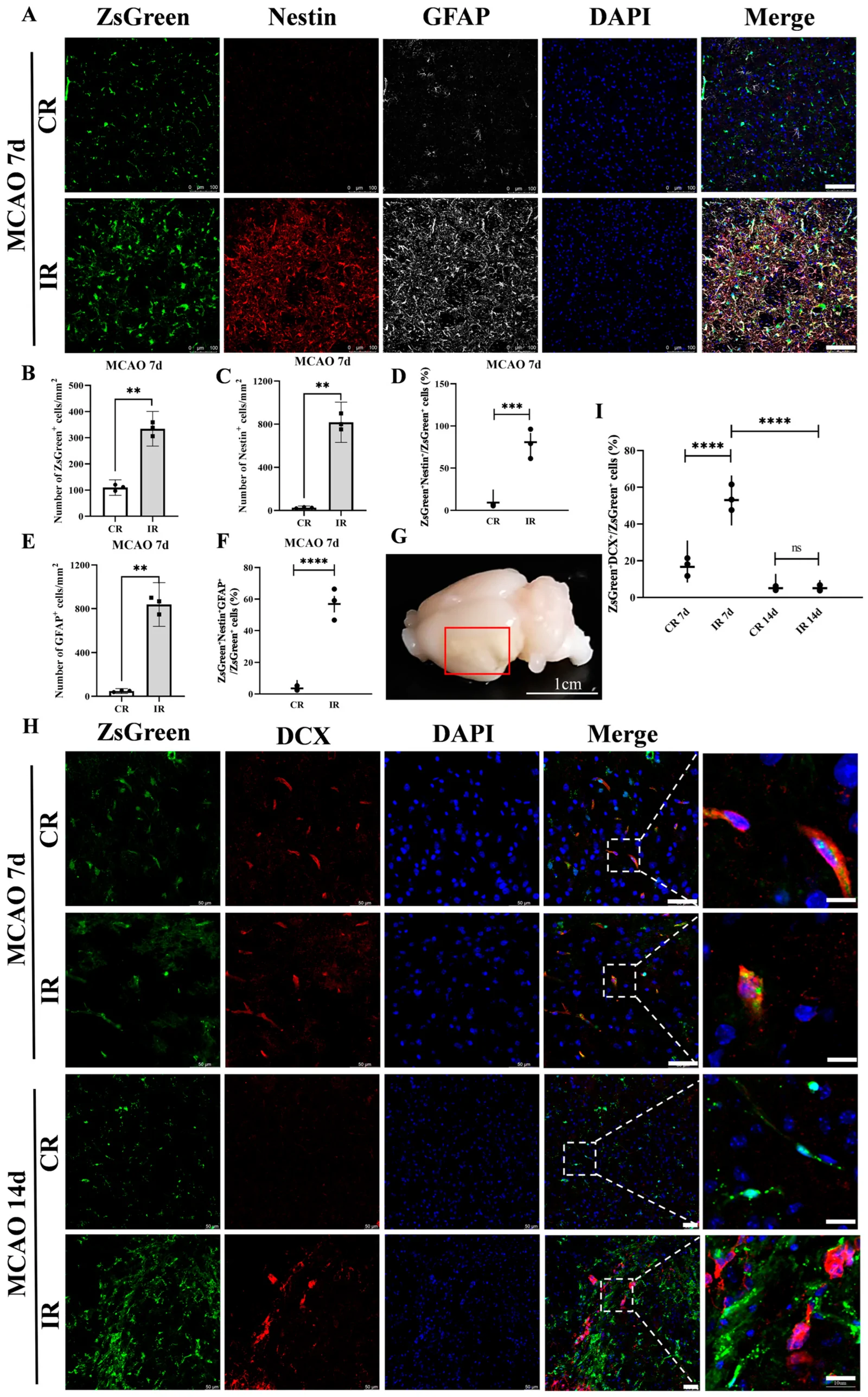
PDGFR-β-lineage cells exhibit temporal changes in Nestin-, GFAP-, and DCX-associated molecular phenotypes after ischemic stroke. (**A**) Representative immunofluorescence images showing ZsGreen, Nestin, and GFAP expression in brain sections from mice at 7 days after MCAO. Color channels are indicated as follows: green, ZsGreen; red, Nestin; grey, GFAP, and blue, DAPI. Scale bars (White thick line): 100 μm. (**B**–**F**) Quantification of ZsGreen-positive cells, Nestin-positive cells, and GFAP-positive cells in MCAO 7d mice shown in (**A**). (**B**) Quantification of number of ZsGreen^+^ cells per mm^2^; t_(2)_ = 26.27, *p* = 0.0014, mean difference = 224.5, 95% CI [187.70, 261.30]. (**C**) Quantification of number of Nestin^+^ cells per mm^2^; t_(2)_ = 17.76, *p* = 0.0032, mean difference = 791.2, 95% CI [599.50, 982.80]. (**D**) Quantification of the percentage of ZsGreen^+^Nestin^+^ cells among ZsGreen^+^ cells. Proportion data were analyzed using beta-binomial regression, with the individual mouse considered the experimental unit. Data were presented as model-estimated proportions with 95% confidence intervals. Estimated difference = 71.65 percentage points, 95% CI [55.57, 87.72], *p* = 0.0007. (**E**) Quantification of number of GFAP^+^ cells per mm^2^; t_(2)_ = 15.85, *p* = 0.0040, mean difference = 790.20, 95% CI [575.60, 1005.00]. (**F**) Quantification of the percentage of ZsGreen^+^Nestin^+^ GFAP^+^ cells among ZsGreen^+^ cells. Proportion data were analyzed using beta-binomial regression, with the individual mouse considered the experimental unit. Data were presented as model-estimated proportions with 95% confidence intervals. Estimated difference = 53.22 percentage points, 95% CI [50.14–56.30], *p* < 0.0001. (**G**) Representative image of the brain tissue from a mouse at 7 days after MCAO. The red box indicates the scar-like region within the injured area. Scale bars: 1 cm (**H**) Representative immunofluorescence images showing ZsGreen and DCX expression in brain sections from mice at 7 and 14 days after MCAO. The white dashed boxes indicate the regions shown at higher magnification; Color channels are indicated as follows: green, ZsGreen; red, DCX; and blue, DAPI. Scale bars (White thick line): 50 μm. The white dashed boxes indicate magnified regions (zoom in 5x); Scale bars (White thick line): 10 μm. (**I**) Quantification of ZsGreen-positive cells and DCX-positive cells in MCAO 7d/14d mice shown in (**H**). (**I**) The proportion of DCX-positive cells among ZsGreen-positive cells was calculated from the corresponding positive and total cell counts. Each dot represents an individual mouse. Proportion data were analyzed using beta-binomial regression with the individual mouse as the experimental unit. Pairwise comparisons were adjusted using the Holm method for multiple comparisons. Data are presented as individual mouse values, with model-estimated proportions and 95% confidence intervals. Data are presented as individual mice with mean ± 95% CI (*n* = 3 mice per time point). Multiple microscopic fields from each mouse were averaged to obtain one value per animal and were not treated as independent biological replicates. Because the IR and CR were obtained from the same animals, comparisons between the two regions (number of cells per mm^2^) were performed using a two-tailed paired Student’s *t*-test. ns, *p* ≥ 0.05, ** *p*  <  0.01, *** *p*  <  0.001, **** *p*  <  0.0001.

At day 7 post-MCAO, the density of ZsGreen^+^ cells was significantly higher in the IR than in the CR (Figure 2A,B). The numbers of Nestin^+^ and GFAP^+^ cells were also increased in the IR compared to the CR; the density of Nestin^+^ cells in the IR exceeded 800 cells/mm^2^ during the subacute phase after ischemic injury (Figure 2A,C,E). Moreover, more than half of the ZsGreen^+^ cells co-expressed Nestin in the IR (Figure 2D). These findings indicate that PDGFR-β-lineage cells exhibit increased expression of Nestin- associated molecular markers during the subacute phase after ischemic injury.

GFAP immunostaining revealed that the density of GFAP^+^ cells in the IR exceeded 800 cells/mm^2^ at 7 days after MCAO than in the corresponding CR (Figure 2A,E). Moreover, more than 40% of ZsGreen^+^ cells co-expressed Nestin and GFAP in the IR (Figure 2F). These findings demonstrate that GFAP expression is increased among PDGFR-β-lineage cells during the subacute phase after ischemic injury. A scar-like structure was also observed within the cortical region of the ischemic hemisphere at 7 days after MCAO (Figure 2G). ZsGreen^+^ cells were detected within and adjacent to this region, indicating a spatial association between PDGFR-β-lineage cells and the injured tissue.

To further define the temporal dynamics of the DCX-associated phenotype, we compared the proportion of DCX-positive cells among ZsGreen-positive cells at 7 and 14 days after MCAO (Figure 2H,I). At 7 days, the proportion was significantly higher in the MCAO group than in the corresponding control group (estimated difference = 43.89 percentage points, 95% CI [28.99, 58.79], Holm-adjusted *p* = 0.0001). In contrast, no significant difference was observed between MCAO and control animals at 14 days (estimated difference = −4.05 percentage points, 95% CI [−10.16, 2.05], Holm-adjusted *p* = 0.177). Importantly, the proportion of DCX-positive ZsGreen-positive cells was significantly lower at 14 days than at 7 days after MCAO (estimated difference = 47.95 percentage points, 95% CI [33.60, 62.30], Holm-adjusted *p* < 0.0001). These findings indicate that the DCX-associated phenotype is transient and is most prominent during the subacute phase after ischemic injury. At 28 days after MCAO, a subset of ZsGreen^+^ cells was observed to co-express the endothelial marker CD31 in the IR (Figure S1A). However, the majority of ZsGreen^+^ cells retained expression of canonical pericyte markers (Figure S1B). These observations indicate that a subset of PDGFR-β-lineage cells exhibits changes in vascular- associated marker expression during the chronic stage after ischemic injury, whereas most lineage-traced cells retain a pericyte-associated phenotype.

### 3.3. KLF2 Knockdown Attenuates OGD/R-Induced Neuronal-Associated Marker Expression in HBVPs

**Figure 3:**
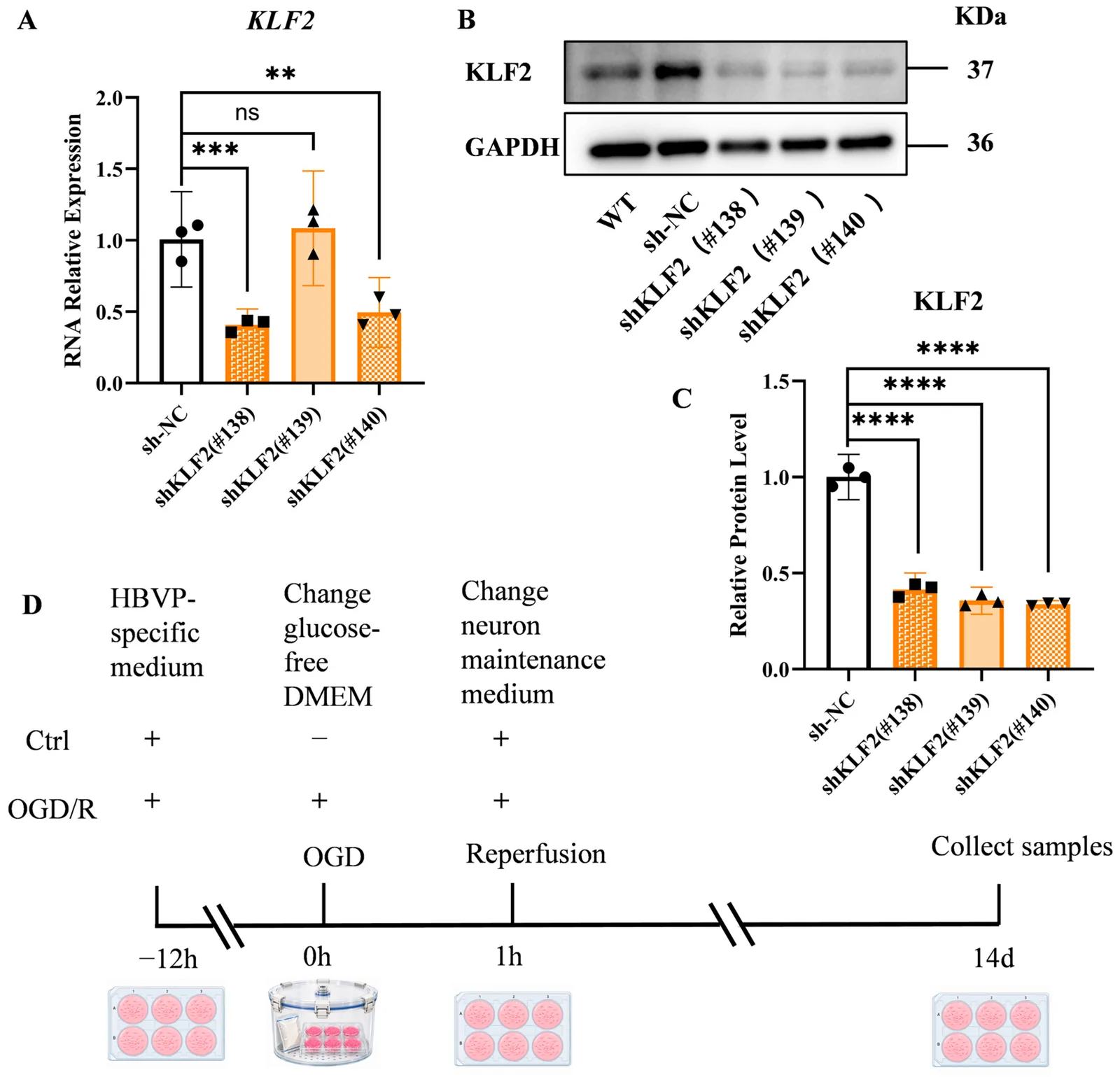
KLF2 knockdown in HBVPs and establishment of the OGD/R model. (**A**) RT-qPCR analysis of *KLF2* knockdown efficiency in HBVPs. sh-NC was used as the negative control, and three independent shRNAs targeting *KLF2* were evaluated: shKLF2 (#138), shKLF2 (#139), and shKLF2 (#140). Statistical significance was determined using ordinary one-way ANOVA followed by Dunnett’s multiple-comparisons test (F_(3,8)_ = 25.72, *p* = 0.0002, η^2^ = 0.90). Compared with sh-NC, *KLF2* expression was reduced by shKLF2 (#138) (mean difference = 0.60, 95% CI [0.32, 0.88], adjusted *p* = 0.0007, q = 6.21) and shKLF2 (#140) (mean difference = 0.51, 95% CI [0.23, 0.79], adjusted *p* = 0.0019, q = 5.29), whereas shKLF2 (#139) did not significantly reduce KLF2 expression (mean difference = −0.08, 95% CI [−0.36, 0.20], adjusted *p* = 0.77, q = 0.80). (**B**) Representative Western blot analysis of KLF2 protein expression following KLF2 knockdown. (**C**) Quantification of KLF2 protein expression shown in (**B**). Statistical significance was determined using ordinary one-way ANOVA followed by Dunnett’s multiple-comparisons test (F_(3,8)_ = 277.4, *p* = 0.0001, η^2^ = 0.99). Compared with sh-NC, KLF2 protein expression was significantly reduced in shKLF2 (#138) (mean diff = 0.59, 95% CI [0.51, 0.66], q = 21.77), shKLF2 (#139) (mean diff = 0.64, 95% CI [0.57, 0.72], q = 23.92), and shKLF2 (#140) (mean diff = 0.66,95% CI [0.58,0.74], q = 24.60) groups (all adjusted *p* < 0.0001). (**D**) Schematic illustration of the OGD/R experimental paradigm in HBVPs. Data are presented as individual biological replicates with mean ± 95% CI (*n* = 3 biological replicates per group). ns: *p*  ≥  0.05; ** *p*  <  0.01; *** *p*  <  0.001; **** *p*  <  0.0001.

KLF2 is an important regulator of vascular homeostasis and cellular responses to environmental stress. However, whether KLF2 contributes to ischemia-associated phenotypic changes in brain vascular pericytes remains unclear. To investigate this possibility, human brain vascular pericytes (HBVPs) were transduced with lentiviral vectors carrying *KLF2*-specific shRNA to suppress KLF2 expression (Figure 3A,B). Among the three shKLF2 constructs tested, shKLF2 (#138) and shKLF2 (#140) produced consistent reductions in *KLF2* mRNA and KLF2 protein levels, whereas shKLF2 (#139) showed insufficient knockdown efficiency at the transcript level. Therefore, shKLF2 (#138) and shKLF2 (#140) were selected for subsequent functional analyses. (Figure 3A–C). An in vitro OGD/R model was subsequently established in HBVPs to evaluate the effects of ischemic–hypoxic stress on pericyte-associated phenotypic responses (Figure 3D). Because neuronal-associated marker expression may require prolonged observation following ischemic stress, cells were analyzed after 14 days of reperfusion following 1 h of OGD treatment, representing a prolonged recovery period after OGD rather than an acute reperfusion paradigm.

**Figure 4:**
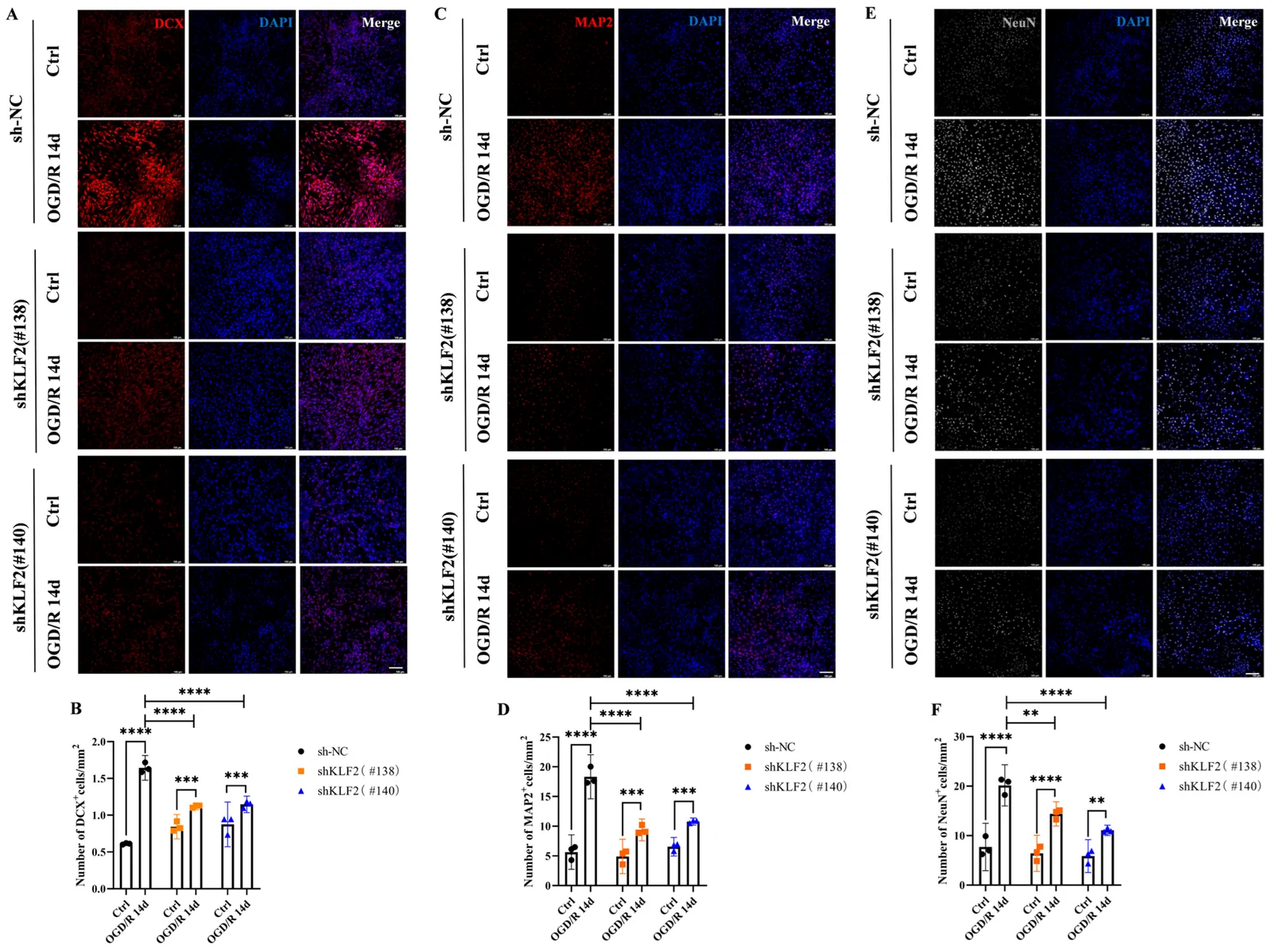
KLF2 knockdown attenuates OGD/R-associated induction of neuronal markers in HBVPs. (**A**,**C**,**E**) Representative immunofluorescence images showing DCX, MAP2, and NeuN expression in HBVPs following OGD/R under the indicated KLF2 knockdown conditions. Color channels are indicated as follows: green, ZsGreen; red, DCX/MAP2; grey, NeuN; and blue, DAPI. Scale bar: 100 μm. (**B**,**D**,**F**) Quantification of DCX-, MAP2-, and NeuN-positive cells shown in (**A**,**C**,**E**), respectively. Data are presented as individual biological replicates with mean ± 95% CI (*n* = 3 independent biological replicates per group). Statistical significance was assessed using two-way ANOVA followed by Sidak’s multiple-comparisons test for prespecified pairwise comparisons. (**B**) Quantification of DCX-positive cells. Because the raw data did not satisfy the normality assumption based on the Shapiro–Wilk test (*p* < 0.05), values were log10-transformed before analysis, after which the transformed data met the normality criterion (*p* > 0.05). Two-way ANOVA revealed a significant interaction between OGD/R treatment and KLF2 knockdown (Interaction F_(2,12)_ = 65.00, *p* = 0.0001, η^2^ = 0.29). Sidak’s multiple-comparisons test showed significant increases in DCX-positive cells following OGD/R in the sh-NC, shKLF2 (#138), and shKLF2 (#140) groups (Ctrl vs. OGD/R 14d. sh-NC: mean diff = −1.03, 95% CI [−1.18, −0.88], t = 19.04, adjusted *p* < 0.0001; shKLF2 (#138): mean diff = −0.28, 95% CI [−0.43, −0.13], t = 5.13, adjusted *p* = 0.0007; shKLF2 (#140): mean diff = −0.27, 95% CI [−0.42, −0.12], t = 5.02, adjusted *p* = 0.0009). Within the OGD/R-treated groups, DCX-positive cells were significantly lower in both shKLF2 (#138) and shKLF2 (#140) groups than in the sh-NC group (OGD/R 14d: sh-NC vs. OGD/R 14d: shKLF2 (#138): mean diff = 0.52, 95% CI [0.33, 0.72], t = 9.63, adjusted *p* < 0.0001; OGD/R 14d: sh-NC vs. OGD/R 14d: shKLF2(#140): mean diff = 0.50, 95% CI [0.30, 0.69], t = 9.15, adjusted *p* < 0.0001). (**D**) Quantification of MAP2-positive cells. Two-way ANOVA revealed a significant interaction between OGD/R treatment and KLF2 knockdown (Interaction F_(2,12)_ = 34.95, *p* = 0.0001, η^2^ = 0.18). OGD/R increased the proportion of MAP2-positive cells in the sh-NC, shKLF2 (#138), and shKLF2 (#140) groups (Ctrl vs. OGD/R 14d. sh-NC: mean diff = −12.68, 95% CI [−14.94, −10.43], t = 15.59, adjusted *p* < 0.0001; shKLF2 (#138): mean diff = −4.470, 95% CI [−6.72, −2.22], t = 5.50, adjusted *p* = 0.0004; shKLF2 (#140): mean diff = −4.243, 95% CI [−6.50, −1.20], t = 5.22, adjusted *p* = 0.0006). Within the OGD/R-treated groups, MAP2-positive cells were significantly lower in shKLF2 (#138) and shKLF2 (#140) groups than in the sh-NC group (OGD/R 14d:sh-NC vs. OGD/R 14d: shKLF2(#138): mean diff = 8.95, 95% CI [5.99, 11.91], t = 11.00, adjusted *p* < 0.0001; OGD/R 14d:sh-NC vs. OGD/R 14d: shKLF2(#140): mean diff = 7.54, 95% CI [4.58, 10.50], t = 9.27, adjusted *p* < 0.0001). (**F**) Quantification of NeuN-positive cells. Two-way ANOVA revealed a significant interaction between OGD/R treatment and KLF2 knockdown (Interaction F_(2,12)_ = 10.37, *p* = 0.0024, η^2^ = 0.08). OGD/R increased the proportion of NeuN-positive cells in all three groups (Ctrl vs. OGD/R 14d. sh-NC: mean diff = −12.44, 95% CI [−15.59, −9.29], t = 10.96, adjusted *p* < 0.0001; shKLF2 (#138): mean diff = −7.95, 95% CI [−11.10, −4.81], t = 7.00, adjusted *p* < 0.0001; shKLF2 (#140): mean diff = −5.19, 95% CI [−8.34, −2.05], t = 4.57, adjusted *p* = 0.0019). Within the OGD/R-treated groups, NeuN-positive cells were significantly lower in shKLF2 (#138) and shKLF2 (#140) groups than in the sh-NC group (OGD/R 14d:sh-NC vs. OGD/R 14d: shKLF2(#138): mean diff = 5.76, 95% CI [1.64, 9.89], t = 7.18, adjusted *p* = 0.0041; OGD/R 14d:sh-NC vs. OGD/R 14d: shKLF2(#140): mean diff = 9.08, 95% CI [4.95, 13.21], t = 11.31, adjusted *p* < 0.0001). ** *p*  <  0.01, *** *p*  <  0.001, **** *p*  <  0.0001.

In sh-NC control cells, OGD/R treatment resulted in increased expression of neuronal-associated markers, including DCX, MAP2, and NeuN (Figure 4A–F). Compared to the ctrl groups, more than three times as many cells in the OGD/R treatment groups exhibited DCX immunoreactivity (Figure 4A,B). In addition, the proportions of MAP2^+^ and NeuN^+^ cells were significantly increased about 10% compared with untreated controls (Figure 4C–F). In contrast, KLF2 knockdown markedly attenuated OGD/R-induced changes in neuronal-associated marker expression. Following OGD/R, the increase in DCX-positive cells observed in sh-NC cells was significantly reduced in shKLF2 (#138) and shKLF2 (#140) groups (Figure 4A,B). Although MAP2 and NeuN expression remained higher than in untreated controls, their induction was significantly lower than that observed in OGD/R-treated sh-NC cells (Figure 4C–F). Together, these findings indicate that KLF2 is associated with the regulation of OGD/R-induced neuronal-associated marker expression in human brain vascular pericytes. However, these changes represent molecular alterations rather than definitive evidence of neuronal differentiation or functional neuronal conversion.

### 3.4. KLF2 Is Associated with OGD/R-Induced ERK1/2 Phosphorylation and MAP2 Expression in Pericytes

**Figure 5:**
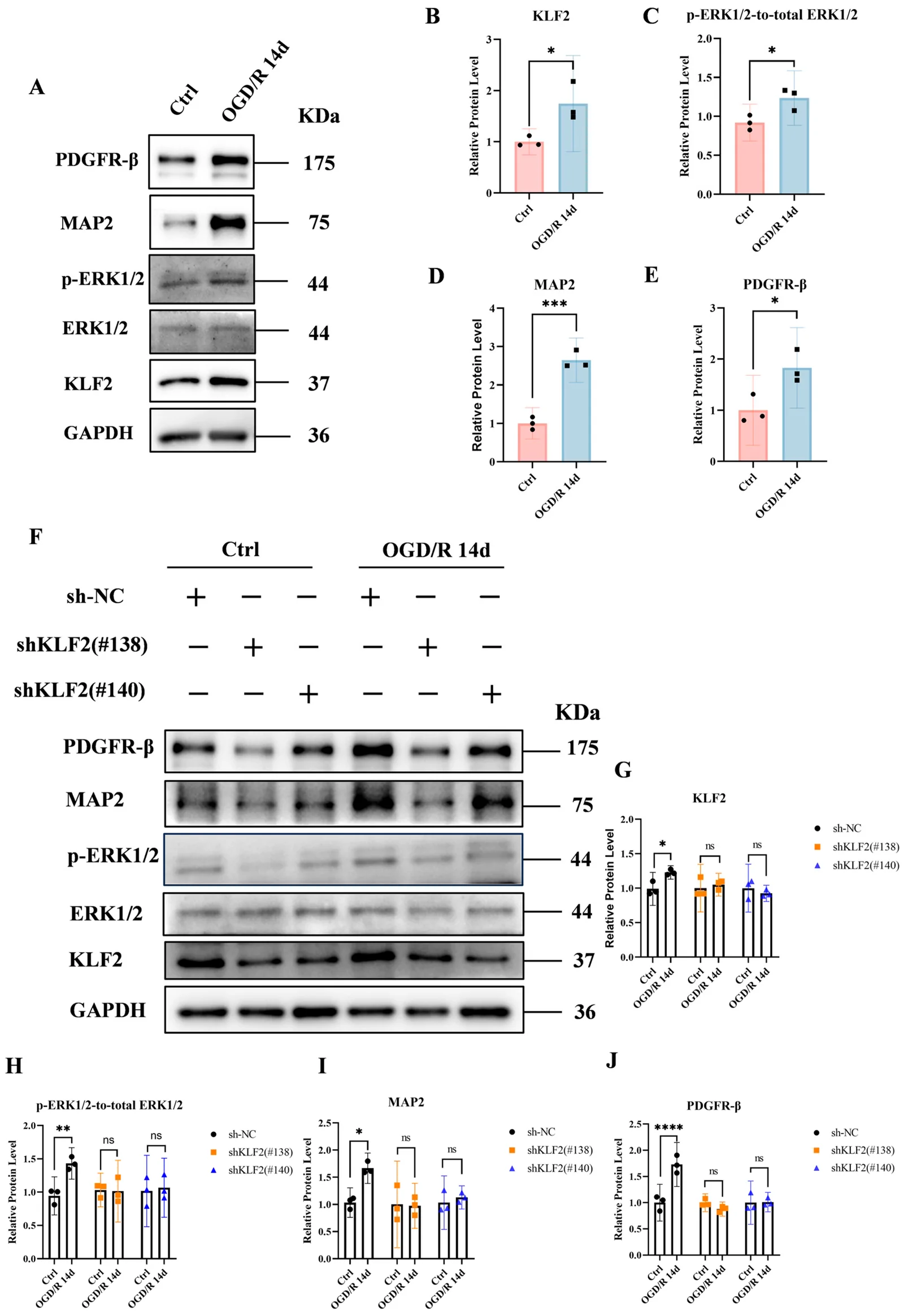
KLF2 knockdown attenuates OGD/R-associated ERK1/2 phosphorylation and MAP2 expression in HBVPs. (**A**) Representative Western blot analysis of KLF2, phosphorylated ERK1/2 (p-ERK1/2), total ERK1/2, MAP2, and PDGFR-β protein expression in HBVPs following OGD/R treatment. (**B**–**E**) Quantification of protein expression shown in (**A**). p-ERK1/2 levels were normalized to total ERK1/2. Data are presented as individual biological replicates with mean ± 95% CI (*n* = 3 independent biological replicates per group). Statistical comparisons were performed using two-tailed unpaired Student’s *t*-tests. (**B**) Quantification of KLF2 protein expression; t_(4)_ = 3.30, *p* = 0.0301, mean difference = 0.75, 95% CI [0.12, 1.37]. (**C**) Quantification of p-ERK1/2 normalized to total ERK1/2; t_(4)_ = 3.23, *p* = 0.0320, mean difference = 0.32, 95% CI [0.04, 0.59]. (**D**) Quantification of MAP2 protein expression; t_(4)_ = 10.01, *p* = 0.0006, mean difference = 1.64, 95% CI [1.19, 2.10]. (**E**) Quantification of PDGFR-β protein expression; t_(4)_ = 3.42, *p* = 0.0267, mean difference = 0.83, 95% CI [0.16, 1.50]. (**F**) Representative Western blot analysis of KLF2, p-ERK1/2, total ERK1/2, MAP2, and PDGFR-β protein expression in HBVPs following OGD/R treatment with or without KLF2 knockdown. (**G**–**J**) Quantification of protein expression shown in (**F**). Statistical analysis was performed using two-way ANOVA followed by Sidak’s multiple-comparisons test. (**G**) Quantification of KLF2 protein expression. Two-way ANOVA showed an interaction effect between OGD/R treatment and KLF2 knockdown (Interaction F_(2,12)_ = 3.967, *p* = 0.0476, η^2^ = 0.398). OGD/R increased KLF2 levels in sh-NC cells but not in shKLF2 cells (Ctrl vs. OGD/R 14d. sh-NC: mean diff = −0.24, 95% CI [−0.46, −0.02], t = 3.01, adjusted *p* = 0.0322; shKLF2 (#138): mean diff = −0.05, 95% CI [−0.27, 0.17], t = 0.64, adjusted *p* = 0.90; shKLF2 (#140): mean diff = 0.07,95% CI [−0.14,0.29], t = 0.95, adjusted *p* = 0.7398). (**H**) Quantification of p-ERK1/2 relative to total ERK1/2. Two-way ANOVA revealed an interaction between OGD/R treatment and KLF2 knockdown (Interaction F_(2,12)_ = 4.632, *p* = 0.0323, η^2^ = 0.44). OGD/R increased p-ERK1/2 levels in sh-NC cells but not in shKLF2 cells (Ctrl vs. OGD/R 14d. sh-NC: mean diff = −0.49, 95% CI [−0.84, −0.13], t = 3.83, adjusted *p* = 0.0072; shKLF2 (#138): mean diff = 0.02, 95% CI [−0.34, 0.37], t = 0.13, adjusted *p* = 0.99; shKLF2 (#140): mean diff = −0.05, 95% CI [−0.40, 0.30], t = 0.39, adjusted *p* = 0.97). (**I**) Quantification of MAP2 protein expression. Two-way ANOVA revealed an interaction between OGD/R treatment and KLF2 knockdown (Interaction F_(2,12)_ = 5.47, *p* = 0.0205, η^2^ = 0.48). OGD/R-induced MAP2 elevation was attenuated following KLF2 knockdown (Ctrl vs. OGD/R 14d. sh-NC: mean diff = −0.63, 95% CI [−1.05, −0.22], t = 4.23, adjusted *p* = 0.0035; shKLF2 (#138): mean diff = 0.03, 95% CI [−0.39, 0.44], t = 0.17, adjusted *p* = 0.99; shKLF2 (#140): mean diff = −0.10, 95% CI [−0.51, 0.32], t = 0.64, adjusted *p* = 0.90). (**J**) Quantification of PDGFR-β protein expression. Two-way ANOVA revealed an interaction between OGD/R treatment and KLF2 knockdown (Interaction F_(2,12)_ = 21.10, *p* = 0.0001, η^2^ = 0.78). OGD/R-associated PDGFR-β elevation was reduced following KLF2 knockdown (Ctrl vs. OGD/R 14d. sh-NC: mean diff = −0.73, 95% CI [−1.01, −0.45], t = 7.32, adjusted *p* = 0.0001; shKLF2 (#138): mean diff = 0.12, 95% CI [−0.15, 0.40], t = 1.22, adjusted *p* = 0.57; shKLF2 (#140): mean diff = −0.01, 95% CI [−0.29, 0.26], t = 0.12, adjusted *p* = 0.99). ns: *p*  ≥  0.05; * *p*  <  0.05; ** *p*  <  0.01; *** *p*  ≥  0.001; **** *p*  <  0.0001.

Previous studies have implicated ERK1/2 signaling in the regulation of MAP2 expression during neural progenitor cell differentiation [26]. In our study, KLF2 knockdown attenuated the OGD/R-induced increases in MAP2-positive cells. We therefore investigated whether KLF2 expression was associated with ERK1/2 phosphorylation and MAP2 expression during the OGD/R-induced phenotypic response of HBVPs. Western blot analysis showed that both KLF2 and p-ERK1/2 (phosphorylated ERK1/2) were upregulated in HBVPs after 14 days of OGD/R treatment (Figure 5A–C), indicating that OGD/R enhanced KLF2 expression and ERK1/2 phosphorylation in HBVPs. Meanwhile, OGD/R treatment increased MAP2 (Figure 5D) and PDGFR-β expression (Figure 5E). Moreover, increased PDGFR-β expression is considered a hallmark of pericyte activation after stroke and traumatic brain injury [28]. These findings indicate that prolonged OGD/R exposure is accompanied by coordinated changes in KLF2 expression, ERK1/2 phosphorylation, and MAP2 expression in HBVPs.

We next examined whether these OGD/R-associated molecular changes were altered following KLF2 knockdown. Under the same OGD/R treatment, knocking down KLF2 reduced the expression of KLF2, p-ERK1/2, MAP2, PDGFR-β that were induced by OGD/R (Figure 5F,G). Despite exposure to the same OGD/R stimulation, KLF2 (Figure 5G) and p-ERK1/2 (Figure 5H) expression in the sh-NC group increased to approximately 1.3-fold of the respective Ctrl levels, whereas MAP2 (Figure 5I) and PDGFR-β (Figure 5J) expression increased to more than 1.5-fold of the corresponding Ctrl levels. In contrast, in the shKLF2 group, the expression levels of p-ERK1/2, KLF2, MAP2, and PDGFR-β following OGD/R remained close to those observed in the corresponding Ctrl group (Figure 5H–J). These findings suggest that KLF2 knockdown is associated with attenuation of the OGD/R-induced changes in ERK1/2 phosphorylation, MAP2 expression, and PDGFR-β expression [29]. Together, these findings demonstrate that KLF2 expression is associated with OGD/R-induced ERK1/2 phosphorylation, MAP2 expression, and changes in PDGFR-β expression in HBVPs. The attenuation of ERK1/2 phosphorylation and MAP2 expression following KLF2 knockdown further suggests that KLF2 may contribute to the molecular response of pericytes to ischemic–hypoxic stress. However, because direct ERK1/2 inhibition, ERK1/2 activation rescue, or KLF2 rescue experiments were not performed, these findings do not establish a direct KLF2 → ERK1/2 → MAP2 regulatory pathway.

## 4. Discussion

In this study, we combined PDGFR-β-CreERT2; ZsGreen lineage tracing and an in vitro OGD/R model to investigate ischemia-associated phenotypic changes in PDGFR-β-lineage pericytes after ischemic stroke. We found that ischemic injury induced rapid Nestin expression in PDGFR-β-lineage cells, followed by transient expression of neuroglial-associated markers, including GFAP and DCX, during the subacute phase. In addition, OGD/R stimulation induced neuronal-associated marker expression and reduced ERK1/2 phosphorylation and MAP2 expression. These findings suggest that KLF2 is associated with ischemia-induced phenotypic remodeling of pericytes and may participate in this process through mechanisms involving ERK1/2 signaling. However, the present findings do not demonstrate definitive lineage conversion or functional neuronal differentiation.

As an important component of the NVU, pericytes not only participate in the maintenance of BBB homeostasis, inflammatory regulation, and cerebral blood flow reconstruction after IS, but also exhibit certain stem cell-like characteristics and lineage plasticity. Increasing evidence has demonstrated that brain pericytes undergo spatiotemporally dependent functional transitions following stroke, predominantly participating in inflammatory responses and vascular injury during the acute phase, while contributing to tissue repair, glial responses, and vascular remodeling during the subacute phase [30,31,32]. However, whether endogenous pericytes represent bona fide multipotent stem/progenitor cells in vivo remains controversial. Although several lineage-tracing studies have suggested stem cell-like properties and multilineage potential of pericytes under pathological conditions, other studies using more stringent genetic fate-mapping approaches have challenged this concept. Guimarães–Camboa et al. [13] reported that some vascular-associated progenitor populations previously considered multipotent may not represent endogenous multipotent pericytes, emphasizing the importance of lineage-tracing strategies and cell-type specificity. Thus, pericytes are better regarded as highly plastic mural cells [33] whose fate transitions are induced by injury-associated microenvironmental cues rather than as constitutive multipotent stem cells.

In the present study, we found that the number of PDGFR-β lineage pericytes within the ischemic injured region was markedly reduced at 1 day after MCAO, whereas Nestin expression was rapidly induced, with the majority of residual ZsGreen^+^ cells expressing Nestin. Subsequently, at 3 days after MCAO, the number of ZsGreen^+^ cells within the injured region increased and was accompanied by a further increase in Nestin expression, indicating an injury-associated activation response in PDGFR-β-lineage cells. These findings are consistent with previous reports showing that pericytes can acquire progenitor-associated molecular features, including Nestin and Sox2 expression, following ischemic or hypoxic stimulation [34,35]. Nevertheless, Nestin expression alone does not establish stem cell identity or multipotency; therefore, these findings should be interpreted as a stemness-associated molecular response rather than definitive stem cell activation.

During the subacute phase after MCAO, a proportion of ZsGreen^+^ cells expressed GFAP and DCX. Since GFAP can be induced in reactive glial cells and other injury-associated cellular states, its expression does not indicate definitive astrocytic differentiation. Similarly, DCX represents a neuroblast-associated molecular feature but is insufficient to demonstrate neuronal lineage conversion. Therefore, these findings suggest transient neuroglial-associated phenotypic remodeling rather than confirmed lineage transition. The reduction in DCX^+^/ZsGreen^+^ cells at 14 days after MCAO may indicate attenuation of this phenotype over time, although changes in marker expression during later stages cannot exclude additional cellular transitions. Further studies integrating transcriptomic and functional approaches are required to clarify the fate of these cells.

Pericytes also contribute to vascular remodeling after ischemic injury through regulation of angiogenesis and vascular stabilization [36]. In this study, a subset of PDGFR-β-lineage cells expressed CD31 at 28 days after MCAO, suggesting acquisition of endothelial-associated characteristics during the chronic phase. However, CD31 expression alone does not prove endothelial lineage conversion. Recent studies have emphasized functional heterogeneity among pericyte populations, with distinct lineage-tracing strategies revealing different injury responses. Loan et al. reported that NG2-tdTomato-labeled pericytes preferentially exhibited neural-associated changes after ischemic injury, whereas Tbx18-tdTomato-labeled pericytes displayed broader injury-induced phenotypic responses, including endothelial-, fibroblast-, and microglia-associated characteristics [33]. These findings highlight that pericyte heterogeneity and experimental lineage definitions may influence the observed plasticity patterns.

The molecular mechanisms regulating injury-associated pericyte phenotypic re-modeling remain incompletely understood. KLF2 is a conserved transcription factor involved in vascular homeostasis [37], inflammatory regulation [30], and endothelial stress re-sponses [38,39]. Although KLF2 has been extensively studied in vascular endothelial cells, its role in pericytes remains largely unclear. Previous studies suggest that KLF2 regulates pathways including PDGF-B/PDGFRβ [10], Notch [40], and TGF-β signaling [41], which are important for maintaining pericyte identity and activation states. Altered KLF2 expression following ischemic stress may therefore influence the adaptive response and phenotypic state of pericytes. In addition, KLF2 has been implicated in neuronal survival and neural development under specific conditions [21,42], suggesting that KLF2 may represent a potential regulatory factor linking vascular stress responses with cellular plasticity. Consistent with this possibility, our in vitro experiments demonstrated that OGD/R induced increased expression of DCX, MAP2, and NeuN in HBVPs. However, these changes should be interpreted as partial acquisition of neuronal-associated molecular characteristics rather than generation of mature functional neurons. KLF2 knockdown reduced DCX-positive cells and attenuated MAP2 and NeuN expression, suggesting that KLF2 is associated with OGD/R-induced neuronal-associated phenotypic responses in pericytes.

The ERK1/2 signaling pathway plays important roles in cell fate regulation, neural development, and neuronal maturation [43]. Previous studies have shown that ERK1/2 activity is associated with MAP2 expression and neuronal differentiation-related processes. In our study, OGD/R treatment increased KLF2 expression, ERK1/2 phosphorylation, and MAP2 expression simultaneously. Furthermore, KLF2 knockdown attenuated ERK1/2 phosphorylation and MAP2 upregulation following OGD/R stimulation. These findings suggest that KLF2 is associated with ERK1/2 signaling changes during ischemia-associated pericyte phenotypic remodeling. However, because ERK inhibition, ERK activation rescue, or KLF2 overexpression experiments were not performed, whether ERK1/2 directly mediates KLF2-associated effects remains unclear. Interestingly, OGD/R also increased PDGFR-β expression, whereas this response was reduced following KLF2 knockdown. Given that PDGFR-β regulates pericyte activation, migration, and vascular remodeling [44], these findings suggest that KLF2 may influence broader stress-associated responses in pericytes. Further studies are needed to clarify the relationship among KLF2, PDGFR-β signaling, and injury-induced phenotypic remodeling.

This study has several limitations. A limitation of this study is the relatively small sample size used in several experiments (particularly those involving only three biological replicates per group), highlighting the need for validation in larger, independently replicated cohorts in future studies. Although PDGFR-β-CreERT2; ZsGreen lineage tracing enabled the longitudinal tracking of PDGFR-β-lineage cells after ischemic injury, ZsGreen labeling indicates lineage origin rather than definitive maintenance of pericyte identity at each time point. Additional characterization using multiple pericyte/mural cell markers and exclusion of other vascular or glial populations will be required to further define the identity and fate of labeled cells after MCAO. The observed neuroglial-associated phenotypes were primarily based on marker expression, including Nestin, GFAP, DCX, MAP2, and NeuN. These findings indicate partial acquisition of injury-associated neuroglial- or neuron-like molecular characteristics but do not establish stable lineage conversion, functional neuronal maturation, or neural repair capacity. Additional approaches, including single-cell profiling, cell sorting, and functional assays, will be required to further characterize these cellular states. The proposed association between KLF2 and ERK1/2–MAP2 signaling was mainly supported by the in vitro HBVP OGD/R model. Although KLF2 knockdown reduced ERK1/2 phosphorylation and MAP2 expression, direct in vivo validation of KLF2, p-ERK1/2, and MAP2 changes in PDGFR-β-lineage cells after MCAO is still required. In addition, cellular viability, proliferation, and cell density were not directly assessed following KLF2 knockdown. Thus, we cannot completely exclude the possibility that alterations in general cellular status contributed to some of the observed changes in neuronal-associated marker expression. The contralateral hemisphere was used as an internal reference in the lineage-tracing experiments; however, ischemic injury may induce bilateral or remote cellular responses. Therefore, future studies incorporating naïve or sham-operated controls will be important to better define injury-specific changes. Finally, further studies using pathway intervention approaches, including ERK modulation and KLF2 rescue strategies, are needed to establish the causal mechanisms underlying pericyte phenotypic remodeling.

## 5. Conclusions

In summary, our findings suggest that ischemic stress induces temporally dynamic phenotypic remodeling in PDGFR-β-lineage cells, characterized by the acquisition of neuroglial-associated molecular signatures during distinct stages after injury. KLF2 may participate in this process through mechanisms associated with ERK1/2 activation and MAP2 expression under ischemic–hypoxic conditions. However, whether these injury-associated phenotypes represent stable lineage conversion or transient adaptive responses remains unresolved. Future studies integrating long-term lineage tracing, single-cell molecular profiling, and functional analyses will be required to determine the fate and biological significance of PDGFR-β-lineage cells during neurovascular remodeling after ischemic injury.

## Data Availability

The raw data supporting the conclusions of this article will be made available by the authors on request.

## Supplementary Materials

The following supporting information can be downloaded at https://www.mdpi.com/article/10.3390/brainsci16090924/s1, Figure S1: CD31-associated vascular phenotypic changes in PDGFR-β-lineage cells after IS in the mice brain. (A) Immunofluorescence staining of ZsGreen and CD31 in brain sections from MCAO 28d pericyte lineage-tracing mice. (B) Immunofluorescence staining of ZsGreen and PDGFR-β in brain sections from MCAO 28d pericyte lineage-tracing mice. Figure S2: Validation of cerebral ischemic injury and blood flow changes following transient MCAO. (A) TTC staining of mice brain tissues subjected to MCAO. (B) Representative LSCI of CBF changes during ischemia and reperfusion. (C) Quantification of cerebral blood flow at different experimental time points. (D) Quantitative analysis of cerebral blood flow changes during ischemia and reperfusion.

## References

[1] GBD 2019 Stroke Collaborators (2021). Global, regional, and national burden of stroke and its risk factors, 1990–2019: A systematic analysis for the Global Burden of Disease Study 2019. Lancet Neurol..

[2] Spitzer D., Guérit S., Puetz T., Khel M.I., Armbrust M., Dunst M., Macas J., Zinke J., Devraj G., Jia X. (2022). Profiling the neurovascular unit unveils detrimental effects of osteopontin on the blood-brain barrier in acute ischemic stroke. Acta Neuropathol..

[3] Liu Q., Zhang X. (2023). Multimodality neuroimaging in vascular mild cognitive impairment: A narrative review of current evidence. Front. Aging Neurosci..

[4] Wang L., Xiong X., Zhang L., Shen J. (2021). Neurovascular Unit: A critical role in ischemic stroke. CNS Neurosci. Ther..

[5] Zheng Z., Chopp M., Chen J. (2020). Multifaceted roles of pericytes in central nervous system homeostasis and disease. J. Cereb. Blood Flow. Metab..

[6] Sepehrinezhad A., Gorji A. (2026). Pericyte-glial cell interactions: Insights into brain health and disease. Neural Regen. Res..

[7] Pham T.T.D., Park S., Kolluri K., Kawaguchi R., Wang L., Tran D., Zhao P., Carmichael S.T., Ardehali R. (2021). Heart and Brain Pericytes Exhibit a Pro-Fibrotic Response After Vascular Injury. Circ. Res..

[8] Li P., Fan H. (2023). Pericyte Loss in Diseases. Cells.

[9] Sato Y., Falcone-Juengert J., Tominaga T., Su H., Liu J. (2022). Remodeling of the Neurovascular Unit Following Cerebral Ischemia and Hemorrhage. Cells.

[10] Sweeney M.D., Ayyadurai S., Zlokovic B.V. (2016). Pericytes of the neurovascular unit: Key functions and signaling pathways. Nat. Neurosci..

[11] Nishiyama R., Nakagomi T., Nakano-Doi A., Kuramoto Y., Tsuji M., Yoshimura S. (2024). Neonatal Brains Exhibit Higher Neural Reparative Activities than Adult Brains in a Mouse Model of Ischemic Stroke. Cells.

[12] Karow M., Sánchez R., Schichor C., Masserdotti G., Ortega F., Heinrich C., Gascón S., Khan M.A., Lie D.C., Dellavalle A. (2012). Reprogramming of pericyte-derived cells of the adult human brain into induced neuronal cells. Cell Stem Cell.

[13] Guimarães-Camboa N., Cattaneo P., Sun Y., Moore-Morris T., Gu Y., Dalton N.D., Rockenstein E., Masliah E., Peterson K.L., Stallcup W.B. (2017). Pericytes of Multiple Organs Do Not Behave as Mesenchymal Stem Cells In Vivo. Cell Stem Cell.

[14] Cho D.I., Ahn J.H., Kang B.G., Hwang I., Cho H.H., Jun J.H., Yoo J., Cho M., Yoo S.J., Kim H.S. (2025). ANGPTL4 Prevents Atherosclerosis by Preserving KLF2 to Suppress EndMT and Mitigates Endothelial Dysfunction. Arterioscler. Thromb. Vasc. Biol..

[15] Wittner J., Schuh W. (2021). Krüppel-like Factor 2 (KLF2) in Immune Cell Migration. Vaccines.

[16] Nayak L., Lin Z., Jain M.K. (2011). “Go with the flow”: How Krüppel-like factor 2 regulates the vasoprotective effects of shear stress. Antioxid. Redox Signal.

[17] Atkins G.B., Jain M.K. (2007). Role of Krüppel-like transcription factors in endothelial biology. Circ. Res..

[18] Boon R.A., Horrevoets A.J. (2009). Key transcriptional regulators of the vasoprotective effects of shear stress. Hamostaseologie.

[19] Zhao Y., He X., Yang X., Hong Z., Xu Y., Xu J., Zheng H., Zhang L., Zuo Z., Hu X. (2025). CircFndc3b Mediates Exercise-Induced Neuroprotection by Mitigating Microglial/Macrophage Pyroptosis via the ENO1/KLF2 Axis in Stroke Mice. Adv. Sci..

[20] Zhu W., Xie N., Li Z., Wang X., Bi K., Zhu K., Dai R., Gao L., Wang Y., Li Y. (2025). Endothelial SRSF1 Promotes Ischemia-Induced Angiogenesis via ATF3-KLF2-S1PR1 Pathway. Circ. Res..

[21] Hansda S., Prateeksha P., Das H. (2024). Krüppel-like factor 2 (KLF2), a potential target for neuroregeneration. Neural Regen. Res..

[22] Iroegbu J.D., Ijomone O.K., Femi-Akinlosotu O.M., Ijomone O.M. (2021). ERK/MAPK signalling in the developing brain: Perturbations and consequences. Neurosci. Biobehav. Rev..

[23] Johnson G.L., Lapadat R. (2002). Mitogen-activated protein kinase pathways mediated by ERK, JNK, and p38 protein kinases. Science.

[24] Wang F., Xia Z., Sheng P., Ren Y., Liu J., Ding L., Yan B.C. (2022). Targeting the Erk1/2 and autophagy signaling easily improved the neurobalst differentiation and cognitive function after young transient forebrain ischemia compared to old gerbils. Cell Death Discov..

[25] Kurtzeborn K., Kwon H.N., Kuure S. (2019). MAPK/ERK Signaling in Regulation of Renal Differentiation. Int. J. Mol. Sci..

[26] Chan W.S., Sideris A., Sutachan J.J., Montoya G.J., Blanck T.J., Recio-Pinto E. (2013). Differential regulation of proliferation and neuronal differentiation in adult rat spinal cord neural stem/progenitors by ERK1/2, Akt, and PLCγ. Front. Mol. Neurosci..

[27] Lei Y., Chen X., Mo J.L., Lv L.L., Kou Z.W., Sun F.Y. (2023). Vascular endothelial growth factor promotes transdifferentiation of astrocytes into neurons via activation of the MAPK/Erk-Pax6 signal pathway. Glia.

[28] Shen J., Xu G., Zhu R., Yuan J., Ishii Y., Hamashima T., Matsushima T., Yamamoto S., Takatsuru Y., Nabekura J. (2019). PDGFR-β restores blood-brain barrier functions in a mouse model of focal cerebral ischemia. J. Cereb. Blood Flow. Metab..

[29] Armulik A., Genové G., Betsholtz C. (2011). Pericytes: Developmental, physiological, and pathological perspectives, problems, and promises. Dev. Cell.

[30] Zhong X., Ye Z., Liu L., Hui Z., Wang S., Zhang A., Zhu G., Zhao F., Li M., Zhang Z. (2026). Spatiotemporal dynamics of brain pericytes in ischemic stroke. J. Neuroinflammation.

[31] Fu J., Liang H., Yuan P., Wei Z., Zhong P. (2023). Brain pericyte biology: From physiopathological mechanisms to potential therapeutic applications in ischemic stroke. Front. Cell Neurosci..

[32] Nakamura K., Ago T. (2023). Pericyte-Mediated Molecular Mechanisms Underlying Tissue Repair and Functional Recovery after Ischemic Stroke. J. Atheroscler. Thromb..

[33] Loan A., Awaja N., Lui M., Syal C., Sun Y., Sarma S.N., Chona R., Johnston W.B., Cordova A., Saraf D. (2024). Single-cell profiling of brain pericyte heterogeneity following ischemic stroke unveils distinct pericyte subtype-targeted neural reprogramming potential and its underlying mechanisms. Theranostics.

[34] Cao L., Zhou Y., Chen M., Li L., Zhang W. (2021). Pericytes for Therapeutic Approaches to Ischemic Stroke. Front. Neurosci..

[35] Sakuma R., Kawahara M., Nakano-Doi A., Takahashi A., Tanaka Y., Narita A., Kuwahara-Otani S., Hayakawa T., Yagi H., Matsuyama T. (2016). Brain pericytes serve as microglia-generating multipotent vascular stem cells following ischemic stroke. J. Neuroinflammation.

[36] Nakagomi T., Kubo S., Nakano-Doi A., Sakuma R., Lu S., Narita A., Kawahara M., Taguchi A., Matsuyama T. (2015). Brain vascular pericytes following ischemia have multipotential stem cell activity to differentiate into neural and vascular lineage cells. Stem Cells.

[37] Shi H., Sheng B., Zhang F., Wu C., Zhang R., Zhu J., Xu K., Kuang Y., Jameson S.C., Lin Z. (2013). Kruppel-like factor 2 protects against ischemic stroke by regulating endothelial blood brain barrier function. Am. J. Physiol. Heart Circ. Physiol..

[38] Oubari H., Berkane Y., Jeljeli M., Lellouch A.G., Smadja D.M. (2025). Engineering the Future of Stem Cells in Vascular Reconstruction: A Leap Towards Functional Endothelialized Tissue-Engineered Vascular Conduits. Stem Cell Rev. Rep..

[39] Saum K., Campos B., Celdran-Bonafonte D., Nayak L., Sangwung P., Thakar C., Roy-Chaudhury P., Owens A.P.I.P. (2018). Uremic Advanced Glycation End Products and Protein-Bound Solutes Induce Endothelial Dysfunction Through Suppression of Krüppel-Like Factor 2. J. Am. Heart Assoc..

[40] Renz M., Otten C., Faurobert E., Rudolph F., Zhu Y., Boulday G., Duchene J., Mickoleit M., Dietrich A.C., Ramspacher C. (2015). Regulation of β1 integrin-Klf2-mediated angiogenesis by CCM proteins. Dev. Cell.

[41] Boon R.A., Fledderus J.O., Volger O.L., van Wanrooij E.J., Pardali E., Weesie F., Kuiper J., Pannekoek H., ten Dijke P., Horrevoets A.J. (2007). KLF2 suppresses TGF-beta signaling in endothelium through induction of Smad7 and inhibition of AP-1. Arterioscler. Thromb. Vasc. Biol..

[42] Wu F., Li C. (2022). KLF2 up-regulates IRF4/HDAC7 to protect neonatal rats from hypoxic-ischemic brain damage. Cell Death Discov..

[43] Lyu J., DeMarco A.G., Sweet R.A., Grubisha M.J. (2025). MAP2 phosphorylation: Mechanisms, functional consequences, and emerging insights. Front. Cell Neurosci..

[44] Divecha Y.A., Rampes S., Tromp S., Boyanova S.T., Fleckney A., Fidanboylu M., Thomas S.A. (2025). The microcirculation, the blood-brain barrier, and the neurovascular unit in health and Alzheimer disease: The aberrant pericyte is a central player. Pharmacol. Rev..

