# Supplementary material for: KLF2 Is Associated with ERK1/2–MAP2 Activation and Neuron-like Phenotypic Remodeling of PDGFR-β-Lineage Cells Following Ischemic Stroke
Source: Brain Sci. 2026 Aug 30;16(9):924. doi: 10.3390/brainsci16090924 (PMC13604145; doi:10.3390/brainsci16090924)

Supplementary Materials:

Figure S1: CD31-associated vascular phenotypic changes in PDGFR-β-lineage cells after IS in the mice brain. **(A)** Immunofluorescence staining of ZsGreen and CD31 in brain sections from MCAO 28d pericyte lineage-tracing mice. Scale bar: 100 μm. **(B)** Immunofluorescence staining of ZsGreen and PDGFR-β in brain sections from MCAO 28d pericyte lineage-tracing mice. Scale bar: 100 μm.

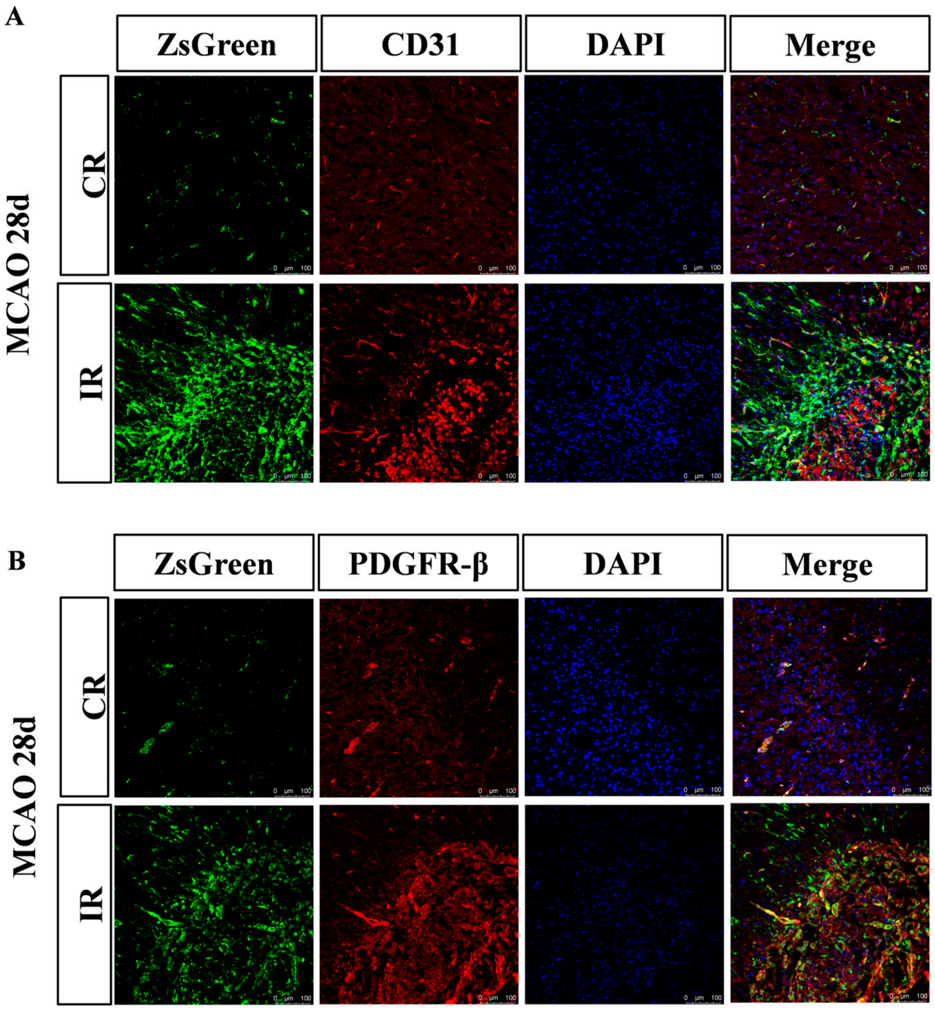

Figure S2: Validation of cerebral ischemic injury and blood flow changes following transient MCAO. **(A)** TTC staining of mice brain tissues subjected to MCAO. White areas indicate ischemic injury regions, whereas red areas represent non-injured tissues. **(B)** Representative LSCI of CBF changes during ischemia and reperfusion. Ctrl, baseline cerebral blood flow before ischemia induction; Ischemia, cerebral blood flow during 30 min MCAO; Reperfusion, cerebral blood flow 1 h after reperfusion following 30 min MCAO. Red color indicates relatively higher blood flow intensity. **(C)** Quantification of cerebral blood flow at different experimental time points. Data are presented as

individual biological replicates with mean ± 95% confidence interval (CI) (n = 5 mice per group). Statistical analysis was performed using ordinary one-way ANOVA followed by Dunnett's multiple-comparisons test ($F_{(2,12)} = 131.5$, $P < 0.0001$, $\eta^2 = 0.96$). Compared with Ctrl, ischemic CBF was significantly reduced (mean difference = −1043, 95% CI [−1207, −877.8], adjusted $P < 0.0001$, q = 15.83). Compared with the ischemic condition, CBF was significantly increased after reperfusion (mean difference = 723.5, 95% CI [558.7, 888.3], adjusted P < 0.0001, q = 10.98). **(D)** Quantitative analysis of cerebral blood flow changes during ischemia and reperfusion. During the 30-min occlusion period, reduction of regional CBF to <20% of the pre-ischemic baseline. Following removal of the filament and reperfusion, cerebral blood flow recovered to >70% of baseline. This result conformed to the criteria for verifying the success of MCAO using LSCI. Data are presented as individual mice with mean ± 95% CI (n = 5 mice per time point). Comparisons between ischemic and reperfusion were performed using a two-tailed paired Student's t-test. Statistical analysis showed a significant recovery after reperfusion (mean difference = 57.83, 95% CI [48.30, 67.34], $P < 0.0001$). ***$p < 0.001$; ****$p < 0.0001$.

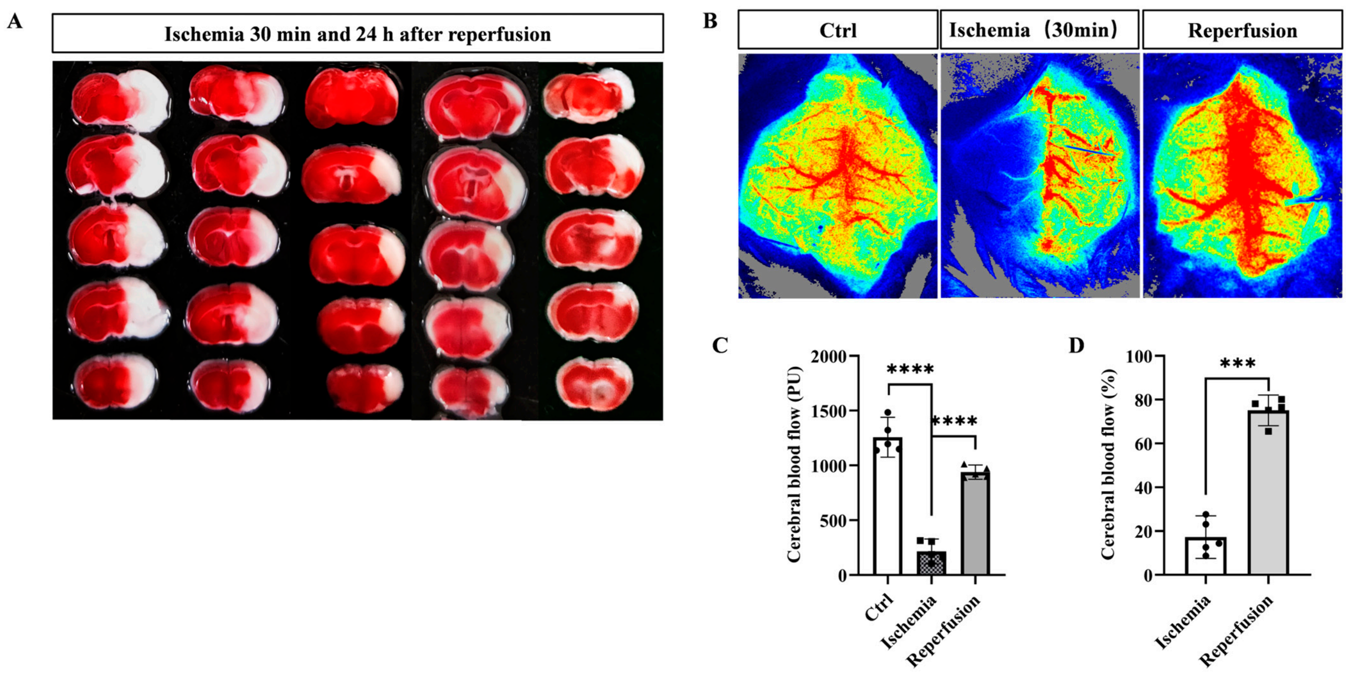

Supplement: Supplementary file 1 [file brainsci-16-00924-s001.zip › brainsci-4441453-supplementary.pdf]
